# Efficacy of alanyl glutamine in nutritional support therapy for patients with sepsis: A protocol for systematic review and meta-analysis

**DOI:** 10.1097/MD.0000000000024861

**Published:** 2021-03-19

**Authors:** Xiaolei Su, Yuemeng Li, Yan Zhang, Shiquan Han

**Affiliations:** Dalian Municipal Central Hospital, Dalian, Liaoning Province, China.

**Keywords:** Alanyl glutamine, nutritional support, protocol, sepsis, systematic review

## Abstract

**Background:**

Sepsis is a systemic inflammatory response caused by infection, which is a common complication after severe infection, trauma, shock, and surgery, and is also an important factor in inducing septic shock and multiple organ dysfunction syndrome (MODS), and has become one of the important causes of death in critically ill patients. Septic patients with gastrointestinal transport function weakened, are prone to malnutrition, resulting in decreased immune function, thereby affecting the therapeutic effect. Clinical practice shows that the nutritional metabolism and immune response of patients with sepsis can be effectively improved by giving alanyl glutamine nutritional support treatment, but there is no evidence of evidence-based medicine. The study carried out in this protocol aims to evaluate the effectiveness of alanyl glutamine in nutritional support therapy for patients with sepsis.

**Methods:**

The Cochrane Library, PubMed, Embase, Web of Science, WHO International Clinical Trials Registry Platform, CNKI, CBM, VIP, and Wanfang databases were searched by computer, to retrieve all randomized controlled trials (RCTs) on nutritional support for the treatment of sepsis with alanyl glutamine from the date of database establishment to December 2020. Two researchers independently selected the study, extracted and managed the data. RevMan5.3 software was used to analyze the included literature.

**Results:**

This study observed the changes of serum albumin (ALB), prealbumin (PAB), hemoglobin (Hb), C-reactive protein (CRP), immunoglobulin (IgG, IgA, and IgM), APACHE II score before and after treatment to evaluate the efficacy of alanyl glutamine in nutritional support therapy for patients with sepsis.

**Conclusion:**

This study will provide reliable evidence for the application of alanyl glutamine in nutritional support therapy for patients with sepsis.

**OSF Registration number:**

DOI 10.17605/OSF.IO/VRZPJ.

## 1 Introduction

Sepsis is a life-threatening organ dysfunction caused by an imbalance in host responses to bacteria, fungi or viral infections.^[1]^ Since there is no specific treatment, sepsis is still one of the most common causes of death for hospitalized patients in intensive care unit (ICU).^[2]^ Epidemiological survey shows that due to aging population, chemotherapy and immunosuppressive therapy widely used and other factors, the incidence of sepsis has steadily increased worldwide,^[3]^ and the high cost of treatment has brought huge economic burden to patients and countries. Therefore, sepsis has become a major public health problem.^[4]^

Sepsis is clinically characterized by increased catabolic rate in patients, massive depletion of glycogen, and lipid reserves,^[5]^ and then a series of metabolic disorders, rapid decline in nutritional status and immune function.^[6]^ Alanyl glutamine, as a conditionally essential amino acid, is one of the special nutrients widely used in clinical practice. It cannot only supplement essential amino acids, but also be an important energy substance for immune cells.^[7,8]^ In recent years, a number of clinical studies have shown that: the application of alanyl glutamine in nutritional support treatment of patients with sepsis can effectively improve the imbalance of nutritional metabolism, protect the damage caused by inflammation, promote the rehabilitation of critically ill patients and shorten the discharge time of patients.^[9–11]^ However, due to the small sample size of the study and the differences in study protocol and efficacy evaluation, the reliability of the results and the promotion of the clinical therapy are affected.

## 2 Objective

In this article, the effectiveness of alanyl glutamine in nutritional support therapy for patients with sepsis was analyzed by meta-analysis. It provides a reliable evidence-based basis for the wide application and scientific research of alanyl glutamine in nutritional support treatment of sepsis patients.

## 3 Methods

### 3.1 Protocol and registration

This protocol of systematic review and meta-analysis has been drafted under the Preferred Reporting Items for Systematic Review and Meta-Analyses Protocols (PRISMA-P) statement guidelines. Moreover, it has been registered in the open science framework (OSF) (registration number: DOI 10.17605/OSF.IO/VRZPJ).

### 3.2 Data sources

#### 3.2.1 Electronic database retrieval strategy

We will use “gu an xian an” (Glutamine), “bing an xian gu an xian an” (alanyl glutamine), “nong du xue zheng” (sepsis), “ying yang zhi chi” (nutritional support), “glutamine,” “alanyl glutamine,” “sepsis,” “pyemia,” “nutritional support” as key words for retrieval, search in 4 Chinese databases, including CNKI, Wanfang, VIP, CBM, and 5 English databases, including PubMed, EMBASE, Web of Science, The Cochrane Library and WHO International Clinical Trials Registry Platform. Taking PubMed as an example, the search strategy is shown in Table 1.

**Table 1:** Retrieval strategy of PubMed.

Number	Search terms
1	Sepsis [MeSH]
2	Pyemia [Title/Abstract] OR Septicemia [Title/Abstract] OR Pyohemias [Title/Abstract] OR Pyaemia [Title/Abstract] OR Blood Poisoning [Title/Abstract]
3	Glutamine [Mesh]
4	Glutamine Dipeptide [Title/Abstract] OR Alanyl Glutamine [Title/Abstract] OR L-Alanyl-L-glutamine [Title/Abstract] OR ALA-GLN [Title/Abstract]
5	Nutritional Support [MeSH]
6	Artificial Feeding [Title/Abstract]
7	1 OR 2
8	3 OR 4
9	5 OR 6
10	7 AND 8 AND 9

#### 3.2.2 Other data sources

In order to obtain the potential non-electronic literature, we will manually retrieve Baidu academic, Google academic, books, impurities, and conference data, in order to obtain information related to the study as comprehensively as possible.

### 3.3 Inclusion criteria

#### 3.3.1 Research type

We will collect all available randomized controlled trials (RCTs) on alanyl glutamine in nutritional support therapy for patients with sepsis, regardless of blinding, publication status, or region. Those studies should be published in English or Chinese before or on December 31, 2020. We will exclude the study not written in English or Chinese due to language bias.

#### 3.3.2 Research object

Patients who met the diagnostic criteria for sepsis proposed by the expert committee of the American College of Chest Physicians and the Society of Critical Care Medicine (ACCP/SCCM 1992),^[12]^ and there is no systemic disease such as blood, immunity, and the corresponding organ function that will affect the experimental results. But the patient's nationality, race, age, gender, course of disease are not limited.

#### 3.3.3 Intervention measures

The control group and treatment group were given conventional anti-infection, nutritional support, symptomatic treatment. The treatment group was added with alanyl glutamine on the basis of the above treatment, and its administration mode, solvent selection, dilution concentration, infusion mode, and infusion speed were unlimited.

#### 3.3.4 Outcome indicators

1. Primary indicators:
  (a) Nutrition index: serum albumin (ALB), prealbumin (PAB), and hemoglobin (Hb) levels;
  (b) Immune index: C-reactive protein (CRP), immunoglobulin (IgG, IgA, and IgM).
2. Secondary indicators:
  (a) APACHE II score: mainly includes acute physiology score (including body temperature, blood pressure, heart rate, respiration, oxygen saturation, arterial blood pH, blood Na+, blood K+, urine volume, and other 13 basic tests), age score and chronic health status score, and the higher the score, the more serious the disease^[13]^;
  (b) Liver and kidney function indexes: ALT, AST, Cr, BUN;
  (c) Length of stay in ICU, ventilator use time, duration of antimicrobial use and incidence of complications (diarrhea and gastric retention).

### 3.4 Exclusion criteria

1. Conference summary, comments, and letters to editors;
2. As for republished literatures, take the one with the most complete data;
3. Literatures with obvious data errors;
4. Literatures without related outcome indicators;
5. Literatures with incomplete data and unable to obtain complete data after contacting the authors;
6. Patients with gastrointestinal dysfunction, diabetes, and renal insufficiency in the study;
7. Patients with digestive tract tumors and previous history of abdominal surgery were included in the study.

### 3.5 Studies selection

According to the predefined inclusion and exclusion criteria, the two researchers independently screened the studies by titles and abstracts, excluded the literatures that did not meet the inclusion criteria, downloaded all the full texts that might be related to the study, and rescreened them by reading the full texts. The two researchers cross-checked the included studies, and any differences were resolved through discussion or participation of a third researcher. The selection process was carried out according to PRISMA flowchart.

### 3.6 Data extraction and management

Two researchers used Excel 2013 software to extract relevant information independently, the contents include:

1. The first author's name and publication years;
2. Basic information of research objects: Sample size, gender, age, and course of disease of the two groups;
3. Intervention measures: the way of administration, concentration, dose, course of treatment of alanyl glutamine;
4. Outcome indicators and specific adverse reactions.

**Figure 1:**
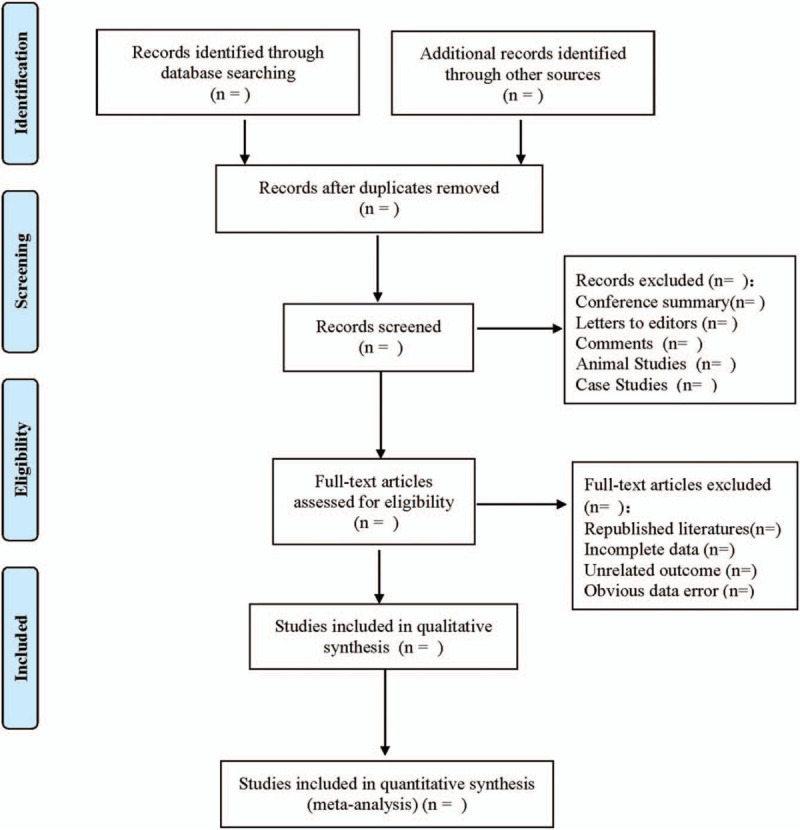
Flow diagram.

If discrepancies occur during data extraction, a third researcher will be involved. The literature screening process is shown in Figure 1.

### 3.7 Dealing with missing data

If the data included in the study are lacking, insufficient or unclear, we will contact the original author by E-mail to obtain more information. If there is no reply from the author or the author has lost the relevant experimental data, we will only analyze the existing data and discuss the potential impact of missing data in the text.

### 3.8 Bias risk assessment

Two researchers will use the Cochrane Reviewers Handbook 5.1.0 recommended tool (Risk of Bias) to independently assess the risk of bias included in the study. The risk of bias assessed includes: random sequence generation, allocation concealment, blinding of participants and personal, blinding of outcome assessment, incomplete outcome data, selective reporting, and other bias. Each item content was to give low (meet all standards)/unclear (lack of judgment information test)/high risk (do not meet any standards) judgment. If there are objections, the third researcher will participate in the assessment.

### 3.9 Evidence quality assessment

We will use the Grading of Recommendations Assessment, Development, and Evaluation (GRADE) system to evaluate the quality of evidence of results. Each result will be evaluated from five aspects: limitations, inconsistency, indirectness, imprecision, and publication bias.^[14]^ The quality of evidence will be rated as “high,” “moderate,” “low,” or “very low.”

### 3.10 Statistical analysis

RevMan5.3 software provided by Cochrane Collaboration Network was used for comprehensive analysis of the extracted available data. For continuous variables, use the mean difference (MD) as the measurement tool if the unit of measurement is the same; if inconsistent, use standard mean difference (SMD). For the binary variables, the relative risk (RR) was used for analysis. The confidence interval (CI) of continuous variables and binary variables was set to 95%.

#### 3.10.1 Evaluation of heterogeneity

Heterogeneity includes statistical heterogeneity, clinical heterogeneity, and methodological heterogeneity. Use *χ*^2^ to do check analysis and to assess the heterogeneity of the included studies combined *I*^2^ quantitative evaluation. If the heterogeneity among studies is low (*I*^2^ < 50%), fixed effect model is used for analysis; if the heterogeneity among studies is significant (*I*^2^ ≥ 50%),^[15]^ the source of heterogeneity should be further analyzed. If it is statistical heterogeneity, the random effect model is used for analysis; if it is clinical heterogeneity and methodological heterogeneity, subgroup analysis or meta-regression analysis are used. If clinical heterogeneity is obvious, but subgroup analysis cannot be performed, descriptive analysis is used.

#### 3.10.2 Subgroup analysis

Subgroup analysis was performed according to different administration methods of alanyl glutamine; Subgroup analysis was performed according to different solvent type of alanyl glutamine injection; Subgroup analysis was performed according to different alanyl glutamine injection dilution concentration; Subgroup analysis was performed according to the use stage and treatment course of alanyl glutamine.

#### 3.10.3 Sensitivity analysis

We will conduct sensitivity analysis on the outcome indicators by excluding each included research one by one, and observe the robustness of the outcome. If necessary, low-quality studies were excluded and repeated meta-analyses were performed to test the stability of the combined results.

#### 3.10.4 Publication bias

When a certain outcome indicator contains 10 or more RCTs, funnel plot was used to evaluate publication bias. If the funnel graph is asymmetric, then explain why the funnel graph is asymmetric, and then use Egger's test and Begg's test to assess potential publication bias.

## 4 Discussion

The pathophysiology of sepsis is complex, which may involve the activation of inflammatory cells and the release of inflammatory mediators, causing secondary intestinal mucosal barrier and immune system dysfunction, translocation of bacteria and endotoxin, resulting in systemic inflammatory response syndrome (SIRS) and multiple organ failure (MOF).^[16,17]^ Because of its sudden strong, rapid development and change of the disease, the prognosis of the sepsis patients is poor and the fatality rate is high.^[18]^

In acute and critical conditions such as sepsis, glutamine in human plasma and tissues is consumed in large quantities and becomes a conditional essential amino acid.^[19]^ Studies have shown that glutamine is not only involved in the synthesis of proteins in the body, but also an important energy material for the proliferation and differentiation of intestinal mucosal cells.^[20]^ Adding glutamine to enteral or parenteral nutrition in clinical patients can reduce the incidence of infectious diseases in some critically ill patients.^[21]^ Since glutamine is easy to hydrolyze and has poor solubility, it is often used in clinical treatment in the form of alanyl glutamine. Alanyl glutamine nutritional support therapy for patients with sepsis can effectively improve their intestinal barrier function and immune function.^[22]^

Studies have shown that glutamine can provide nutritional support for lymphocytes, especially increasing the number of T cells and B cells in vivo. The addition of alanyl glutamine in nutritional supplements can protect the normal structure and function of intestinal mucosa, and even stimulate the intestinal tract to produce more IgA.^[23,24]^ In addition, alanyl glutamine can also maintain the antioxidant reserves in tissues, which has the effects of anti-lipid peroxidation and protection of superoxide dismutase (SOD) activity.^[25]^ Based on this, in the treatment of critically ill patients such as sepsis, giving alanyl glutamine nutritional support therapy can reduce mortality and shorten hospitalization time.^[26]^ However, the role of alanyl glutamine in nutritional support for patients with sepsis has not been recognized by authoritative international medical organizations. Therefore, it is necessary to analyze the results of RCTs of alanyl glutamine nutritional support in the treatment of sepsis, so as to provide a reliable basis for the clinical application of this nutritional support therapy. However, there are still some limitations in this study. Due to language limitations and the quality of the original research included, the accuracy of the results of this study may be biased.

## Author contributions

**Data curation:** Xiaolei Su, Yuemeng Li.

**Funding acquisition:** Shiquan Han.

**Resources:** Yuemeng Li, Yan Zhang.

**Software:** Shiquan Han.

**Supervision:** Yan Zhang.

**Writing – original draft:** Xiaolei Su, Yuemeng Li.

**Writing – review & editing:** Xiaolei Su, Shiquan Han.
